# Elements of Surgery

**Published:** 1833-04-01

**Authors:** 


ELEMENTS OF SURGERY. By *Robert Liston*, Surgeon to the Royal Infirmary, &c. &c. Part Third. Octavo, pp. 409. London and Edinburgh, 1832.

Two parts of Mr. Liston's work have been already published, and were introduced at the time of their publication to the notice of our readers. Being of an elementary character, we cannot pretend to review it, but we think we may venture to give a general idea of its merits by a glance at one or two chapters.

### AFFECTIONS OF THE RECTUM.

The following short and accurate account of the anatomical structure of hæmorrhoidal tumours is not unworthy of notice. After stating that branches of the hæmorrhoidal veins become varicose, probably from their superior trunks being compressed, Mr. L. proceeds to observe :—

> "The varix protrudes the superimposed mucous membrane ; and at first the excrescence is composed of the dilated venous trunks containing fluid blood, and invested by the membrane, which inflames, thickens, loses its villous character, and discharges a vitiated secretion. In this stage the tumour is easily compressible, and by pressure may be made to disappear almost entirely, the communications between the varicose vessels and the trunks above being still unobstructed. But inflammatory action is soon kindled in the incommoded veins, as frequently happens in varix of the lower extremities; their coats become changed, are thickened, effuse lymph externally and internally, adhere to one another, and are ultimately matted into one confused and solid mass; the contained blood coagulates, becomes fibrinous, the whole tumour feels hard and firm, and often is painful. At length all traces of venous structure disappear; the tumour seems to consist chiefly of effused lymph, condensed cellular tissue, and coagula.
>
> In not a few instances, however, the contents of the veins remain partially fluid, and a communication exists between the vessels of the tumour and those of the surrounding parts.
>
> That such is the usual structure of piles, I am convinced from repeated and careful dissection of the tumours." 64.

Mr. Liston's treatment of piles is no novelty, but evinces judgment. In slight cases he employs astringent ointments or decoctions, with sedulous attention to cleanliness—for inflamed tumours, abstraction of blood by leeches or punctures, and subsequently fomentations—when constriction by the sphincter has occurred, replacement of the tumour, if possible—for irritability of the sphincter, a bougie or incision—for internal piles, when an operation is required, the ligature, and for external, excision.

*Inflammation of the Rectum.* This may follow hæmorrhoids, or the operation for them, or may be occasioned by ascarides, or by hardened fæcal collections or biliary concretions in the cavity of the bowel, or foreign substances, as bones of small animals, needles, pins, &c. irritating or wounding its coats. Mr. Liston describes the symptoms of inflammation of the rectum thus:

"It is attended with excruciating pain, burning heat, and a feeling of contraction, increased very much when the parts are thrown into action by evacuation of the contents of the bowel, or of the bladder. The heat may be felt on introducing the finger, with the view of examination; by doing so, dreadful torture is produced, and such manipulation should not be had recourse to unless there is a suspicion of foreign matter lodging in the part, by removal of which the action might be cut short. The bladder is often affected sympathetically; there may be frequent desire to empty it, or else retention of its contents; this latter occurrence not unfrequently follows operations on the bowel, as for the removal of hæmorrhoids. The inflammation extends to the cellular tissue round the rectum, with swelling and increased pain; the pain is aggravated by pressure, and the patient is unable to sit erect. As the painful symptoms abate, puriform discharge from the membrane of the gut takes place, and often is very profuse. The morbid action sometimes extends to the other intestines, attended after a time with mucous or even bloody evacuations. When the affection is confined to the rectum, the fæces and vitiated secretion are distinct from each other, and the former are usually of their natural appearance; but when the other intestines participate, to a greater or less extent, the fæces are fluid, and intimately mixed with the morbid secretion.

Ulceration of the mucous coat, with continued discharge, often supervenes. Sometimes the peritoneal coat of the bowel is affected secondarily, and then the pain is much more acute and more aggravated by pressure." 67.

Effusion, succeeded by suppuration, takes place in the cellular membrane round the gut, attended with fever of greater or less severity, according to circumstances. In persons of bad habit, or under unfavourable circumstances, the collection of matter may be most extensive, and productive of the worst form of typhus. Several cases of this kind have been recorded. A patient, for instance, has all the symptoms of typhus gravior without apparent cause, when by accident, or by careful examination, an abscess is discovered in the neighbourhood of the anus; it is punctured, and a fishbone escapes.

Mr. Liston also describes a carbuncular affection of the skin and cellular tissue in the vicinity of the anus. There is partial suppuration and extensive sloughing of the cellular tissue, and the symptoms of depression soon become severe, and often fatal. Free and early incisions and stimulants alone can save the patient.

For inflammation of the rectum Mr. Liston recommends antiphlogistic treatment, local or general, according to circumstances—leeches and fomentations to the perinæum—and if suppuration occur here, an early opening. We recollect a remarkable case. A woman had a small circular ulcer of the rectum, just above the external sphincter. After an examination of it fever set in, and some tenderness of the abdomen, with sickness, succeeded. The case appeared to be one of muco-enteritis. At the end of five or six days the woman died. On examination of the body the ulcer of the rectum was found not to have passed quite through the muscular tunic.

Inflammation and deposition of lymph had taken place between the muscular and mucous coats, and had extended up between the same tunics throughout the intestinal canal; there was comparatively little peritoneal inflammation.

"It has been recommended that, in abscess extending along the gut, the cavities of the bowel and abscess should be at once laid into one by incision. I have done so, but always found the cure to be tedious. It is better that the matter should first be evacuated through an external opening, that the painful symptoms and constitutional disturbance should be allowed to subside; and that after the cavity has contracted, and the extent of the sinus been ascertained, the operation should be performed." 81.

In considering *fistula ani*, Mr. Liston adverts to the means of detecting an internal fistula, that is, one which opens into the intestine, but does not open externally. He remarks that we may suspect its existence, when we observe puriform discharge from the bowel, increased on going to stool, and then accompanied with tenesmus; pressure on the side of the anus causing pain, and sometimes an augmentation of discharge; and in many instances hardness, deeply seated. On introducing the finger into the rectum, the aperture in the coats of the bowel is perceived, or a part of the bowel feels more boggy and tender than the rest; through this point a curved probe, introduced along the finger, may be passed into the sinus, and being then directed downwards, reaches the outer extremity of the canal, causes the integuments to project, or is readily felt from the surface. The internal opening is usually immediately within the sphincter, seldom higher. The discharge, in general, is rather profuse, the bowel is very irritable, desire to evacuate it is frequent, and the fæces are often tinged with blood. There is a sensation of itching about the fundament, the heat of the parts is felt by the patient to be increased, he is unable to bear pressure there, and sits on one hip: in most cases the bladder sympathises considerably. Mr. Liston strongly recommends the use of the speculum in these cases, as an adjuvant to the sense of touch. By these means, the surface of the bowel, for five or six inches above the anus, can be examined "as accurately as if it were an external part."

Mr. Liston relates a case, in which a portion of bougie remained for a length of time at the bottom of a fistula, and produced very troublesome consequences. As the case is brief, we will extract it.

*Case.* "A middle-aged man, when in Holland, laboured under a very deep and extensive fistula in ano. Sinuses were divided in all directions, and some healed; one, however, remained open, leading towards the gut from near the tuberosity of the ischium on the left side. He was desired to keep this open by means of bougies, which, as many were used, he manufactured himself out of cloth and plaster. On one occasion, a portion passed deeply, and could not be extracted; but his alarm at this occurrence was appeased on being told that the foreign body would be absorbed. His condition at that time was very miserable; and inflammation was often excited in the parts, with fresh collections of matter. At the same time, he laboured under stricture of the rectum and urethra. He applied to me fifteen years after the commencement of the disease. Then the most troublesome symptom was a constant itching in the perineum, and round the anus, preventing sleep, and causing much excoriation from involuntary scratching; besides, he was annoyed by seminal emissions, and frequent desire to make water. I first divided a small internal fistula, and some time afterwards operated on a large complete one; in the latter instance, a foreign body was felt deep in the wound, the incision was extended, and a large portion of bougie, firmly impacted, was with some difficulty withdrawn. Some days after, other portions of bougie were extracted along with hairs; and these continued to be discharged for many weeks. The symptoms were much relieved. An occasional itching remained, but disappeared after the cure of a very bad stricture in the urethra. He recovered perfectly from the complication of diseases." 75.

Whilst adverting to the treatment of malignant disease of the rectum, Mr. Liston makes a remark which is calculated to excite no small share of surprise and disgust. Female patients, says he, have by some been cruelly treated; the vagina and diseased bowel have been laid into one loathsome cavity, and though the patients have continued to pass excrement and discharge through this cloaca, with the symptoms undiminished, themselves miserable, and obnoxious to others, still such cases have been reported as cures! Can such things be? Is it possible that Mr. Liston has witnessed such daring charlatanerie?

We will now take the liberty of passing to another subject—stone in the bladder. Mr. Liston is well known to be a most dexterous, and a very successful operator; his opinions on lithotomy are, therefore, entitled to some attention.

Mr. L. observes that he has, on several occasions, removed from children concretions of considerable size through the urethra, by means of properly constructed forceps. He prefers Weiss's. He has experienced no difficulty in operating with it in many cases, and at all periods of life.

Mr. Liston appears to be no great friend to lithotrity or the lithotritists. He employs rather sharp expressions. In turning, says he, to the records of *Lithotrity*—and under this term we shall include all attempts to break down stones within the bladder, whether by drilling, or filing, or hammering—it will be found that many patients have died from the mere exploration; and altogether, nearly a half of those who have fallen into the hands of the experimenters and adventurers have perished in consequence. Every successful case is well advertised; the dead men rest in peace.

Mr. L. recommends all surgeons to make themseves sufficiently acquainted with the lithotritic instruments to be able to employ them if necessary. He prefers the three-branched lithotrite of Civiale.

"Various forms of drill have been contrived for acting on a large surface of the stone; others for scooping it out, the shell to be afterwards broken into fragments and triturated; they are unsafe and ineffectual. The instrument is also so constructed that a drill-bow may be used, and the apparatus may be fixed by what mechanics call a bench, or it may be attached, by complicated machinery, to the table on which the patient is laid, and be there secured in a proper position. But all this implies an intention of attacking large and dense stones, and a repetition of the attempts. So far as my experience goes,—and besides having seen Civiale and others operate, I have myself employed the instruments in a good many cases, and in some successfully,—I should dissuade from all endeavours to rid the patient of stone by such means, unless its size and consistence were such, that it would yield to one or two attacks, and to the drill set in motion by the fingers." 193.

Mr. Liston criticises severely M. Heurteloup's plan of breaking the stone by the stroke of a hammer. It certainly does appear to be a dangerous experiment. In point of fact, Mr. Liston does not believe that lithotrity will ever supersede lithotomy. We cannot afford space for Mr. L.'s mode of performing the latter operation, but we can advert to one or two points. In alluding to hæmorrhage from the artery of the bulb, Mr. L. observes that, in his own practice, he never but once had hæmorrhage, chiefly, he believes, from never cutting *upwards* after the first incision. The knife which Mr. L. employs, has a blade and handle somewhat longer than those of a common dissecting knife, and without an edge till within an inch and a half from the point. With respect to the prostate, Mr. Liston's opinions appear to coincide pretty closely with those of Mr. Brodie. The latter able surgeon maintains that the capsule of the prostate should never be divided, and that, therefore, it is better to dilate or tear the prostate than to cut it extensively. Mr. Liston directs the knife to be carried forwads through the prostate, with the edge directed downwards and outwards, cutting the gland obliquely. The knife is to be raised very little from the groove, the object being to divide the gland to the extent of no more than barely three quarters of an inch. By so doing, says Mr. L. the reflection of the pelvic fascia remains uninjured, and the boundary is entire betwixt the external cellular tissue, and that loose and very fine texture immediately exterior to the bladder—betwixt it and the fascia immediately lining the pelvis.

"Some stones are of such a size, as will not admit of passage through the section of one side of the gland. By using the blunt-pointed knife, directed by the finger, without any additional external incision, a wound is made on the right side of the prostate, in the same direction and to the same extent as that on the left. Thus a triangular flap is formed, the apex towards the membranous portion of the urethra, and through the opening thereby afforded, any stone which will pass through the bones of the pelvis can be extracted without much difficulty. But no benefit can result from cutting both sides of the prostate, either by the double lithotome or in the manner just detailed, in all cases. It is time enough to incise the opposite side, when, by introduction of the finger through the usual wound, it has been ascertained that the stone is too large to pass through. Then it is safer to cut the other side, than to enlarge the original opening, either by the knife, or by laceration in cruel attempts to extract the stone through an insufficient opening." 204.

Mr. Liston is a decided advocate for the introduction of a gum-elastic catheter into the bladder through the wound, after the performance of the operation. The catheter is to be secured in its situation. For some hours after the operation it is necessary to clean out the instrument frequently with a feather, to prevent its extremity from becoming obstructed with coagula. After the operation Mr. L. gives diluents copiously, bland nourishment sparingly. Depletion, he remarks, is seldom required, danger not being so likely to occur from peritoneal inflammation, as from urinous infiltration of the cellular tissue.

"In the fatal cases, unconnected with hæmorrhage or exhaustion, the peritoneum is not found vascular or coated with lymph, nor is there collection of morbid secretion from this membrane within the abdominal cavity, but the cellular tissue, along the track of the wound, is black, disorganised, easily lacerable, putrid; or, if the infiltration has not been to such an extent or in such a site as to kill speedily as if by poisoning, unhealthy suppurations are found, extensive, uncircumscribed, composed of sanies, urine, and dead cellular tissue, horribly mixed. Should fixed and increasing pain be complained of in the hypogastrium, the part is to be leeched and fomented; this is the only indication of inflammatory action which has occurred in any of my patients, and it has yielded to the simple treatment here mentioned ; so far as I recollect, in only three cases was the leeching necessary. Some patients require support very soon, almost from the first ; others evince sufficiency of action throughout, and in them it is very necessary to pay strict attention to the state of the stomach and bowels, lest the action should exceed ; some proceed favourably for a time, and then become torpid and stationary, their spirits and constitutional power flagging, in consequence of confinement and the discharge and irritation of the wound,—such also require judicious support, and perhaps slight stimulation." 209.

Mr. Liston has only performed the *recto-vesical* operation once, and that under peculiar circumstances. The patient was aged 64, the symptoms had been irregular in their appearance. On introducing the finger into the rectum a firm, globular, large, and very slightly moveable substance was felt, and on sounding it was evident that the stone struck by the instrument was connected with this body. The lateral operation was performed, and both sides of the prostate divided. The stone could be easily laid hold of, but not moved. On examination it was found that the stone was firmly enveloped by a cyst, situated between the rectum and posterior part of the prostate, and that only a small part projected into the bladder. Mr. L. laid the bowel, cyst, and track of the wound into one cavity by cutting the upper and anterior part of the cyst, passing a blunt-pointed and curved bistoury behind the remainder of the cyst, insinuating it through the coats of the gut at that part, meeting the point with the fore-finger of the left hand passed per anum, and then carrying the instrument forwards to the surface. The stone was easily removed by a strong curved scoop. Some superficial sloughing took place in the wound, but the patient appeared to be doing well, when, at the end of the fifth week he was seized with vomiting and purging, which lowered him very much. The wound made no progress, sloughing of the back took place, and he sank about the end of the eighth week.

We have already exceeded our limits. We think, however, that the extracts we have made, and the notice we have offered of the volume before us, will tempt many of our surgical brethren to consult it themselves.

